# Flecainide fallout: a rare case report of refractory ventricular tachycardia and updated management strategies

**DOI:** 10.1093/ehjcr/ytaf368

**Published:** 2025-07-29

**Authors:** Abhishek Kumar, Manisha Gupta, Amratansh Varshney, Rounak Kumar

**Affiliations:** Department of Cardiology, Government Super Speciality Hospital CIMS, Koni, Bilaspur, Chhattisgarh 495009, India; Department of Neurology, DKS Postgraduate and Research Institute, Ghadi Chowk, Raipur, Chhattisgarh 492002, India; Department of Cardiology, All India Institute of Medical Sciences, Tatibandh, Raipur, Chhattisgarh 492009, India; Department of Cardiology, All India Institute of Medical Sciences, Tatibandh, Raipur, Chhattisgarh 492009, India

**Keywords:** Flecainide, Arrhythmia, Sodium channel blockade, Wide complex tachycardia, Sodium bicarbonate, Case report

## Abstract

**Background:**

Flecainide is a Class IC antiarrhythmic drug used to treat arrhythmias such as atrial fibrillation (AF), paroxysmal supraventricular tachycardia, and ventricular tachycardia (VT). Its mechanism involves blocking sodium channels, leading to QRS widening, especially at higher heart rates. This property increases the risk of pro-arrhythmic events, particularly in patients with structural heart disease or ischaemia.

**Case summary:**

A 67-year-old woman receiving flecainide for AF suffered an ischaemic stroke and later developed VT resistant to amiodarone and direct current cardioversion. Her condition improved significantly after intravenous sodium bicarbonate was administered, which counteracted the effects of sodium channel blockade. Later, she was found to have hyponatremia and slightly elevated flecainide values above the upper limit. This case highlights the complexities of flecainide therapy and the importance of timely intervention in managing arrhythmias.

**Discussion:**

This case underscores the arrhythmogenic potential of flecainide. The development of VT suggests that flecainide may have created a substrate for arrhythmias due to its effects on cardiac conduction. The successful use of sodium bicarbonate illustrates an effective treatment strategy for reversing flecainide-induced toxicity. Clinicians should remain vigilant and consider alternative therapies in managing patients at risk for arrhythmias while on flecainide.

Learning points

- *Pro-arrhythmic potential*: Managing patients on flecainide necessitates vigilant monitoring due to its potential to induce life-threatening ventricular tachycardia, requiring continuous electrocardiogram assessment of QRS duration and readiness to intervene for arrhythmias.
- *Pre-treatment evaluations*: Key strategies for safe flecainide use include conducting pre-treatment evaluations for coronary artery disease and atrioventricular node dysfunction to identify patients at higher risk for adverse effects.
- *Sodium bicarbonate administration*: Prompt administration of sodium bicarbonate is crucial for effectively managing wide complex tachycardia induced by flecainide, as it reverses sodium channel blockade and restores normal cardiac conduction.

## Introduction

Flecainide is an antiarrhythmic agent that belongs to Class IC (reducing Phase 0 slope of the fast sodium channels) and has long been favoured for rhythm control in patients with atrial fibrillation (AF) due to its efficacy in preventing recurrences.^1^ However, flecainide’s use is not without risks. Brugada *et al*.^2^ have emphasized that while flecainide is effective in patients without structural heart disease, it modifies impulse propagation in thin myocardial layers, facilitating re-entry induction. This effect can result in life-threatening arrhythmias in approximately 3% of cases. However, in patients with structural heart disease, ischaemia, or prior myocardial infarction, flecainide increases arrhythmic mortality risk by 2.5-fold. Because of a narrow therapeutic index of flecainide (0.2–1.0 µg/mL), even small fluctuations in its blood concentration can result in life-threatening adverse effects.^3^

## Summary figure

**Figure:**
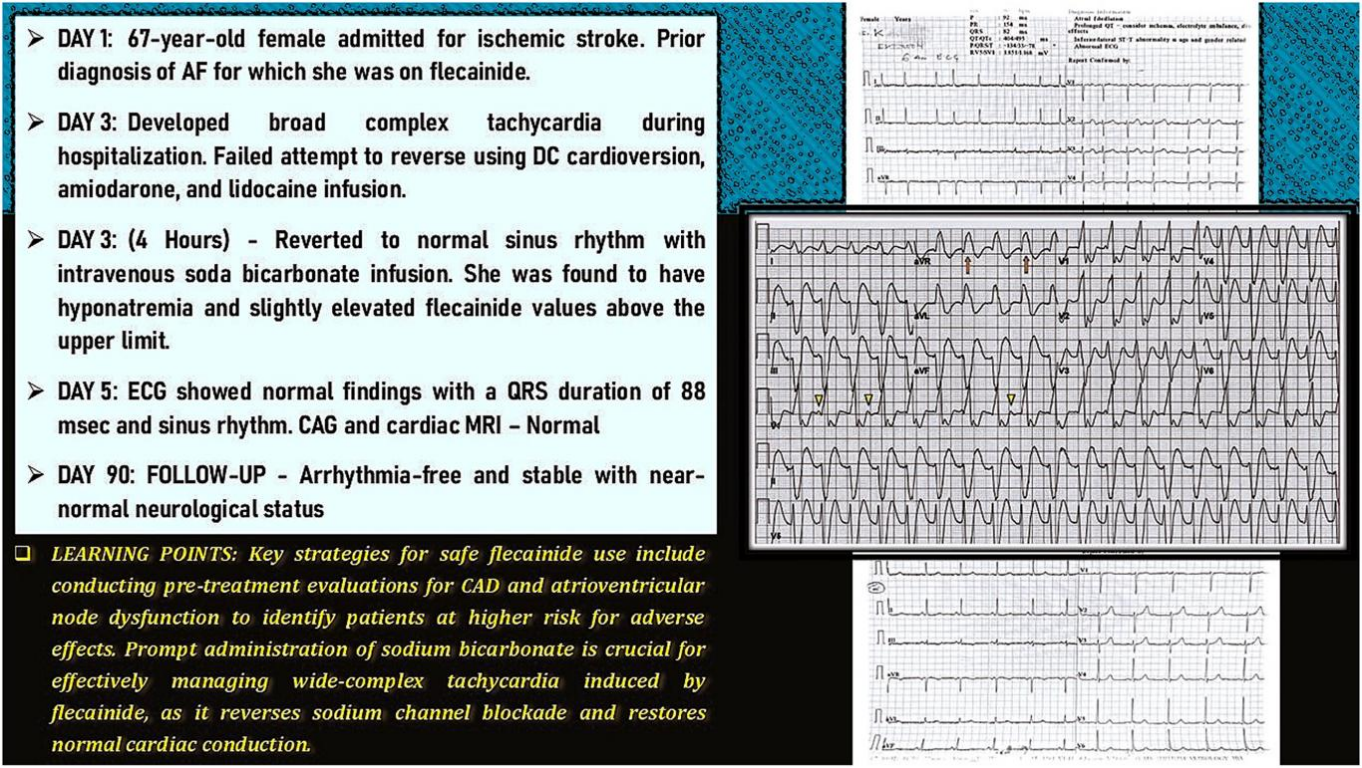


## Case presentation

**Figure 1:**
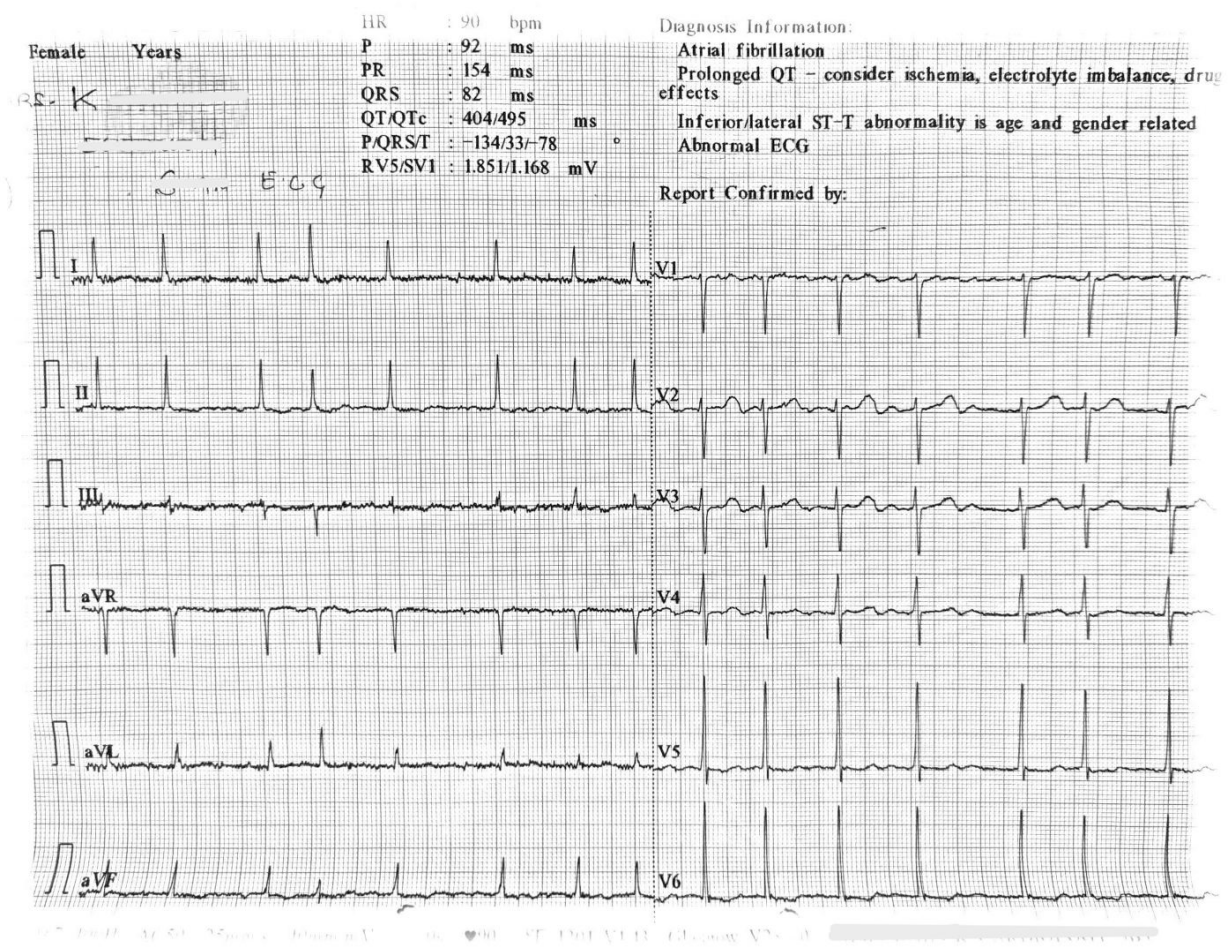
An electrocardiogram of the patient at the time of admission showing an irregular rhythm with narrow complex QRS wave with absent P waves, indicating atrial fibrillation at a controlled ventricular rate of 90 b.p.m. and a QTc interval of 495 ms.

**Figure 2:**
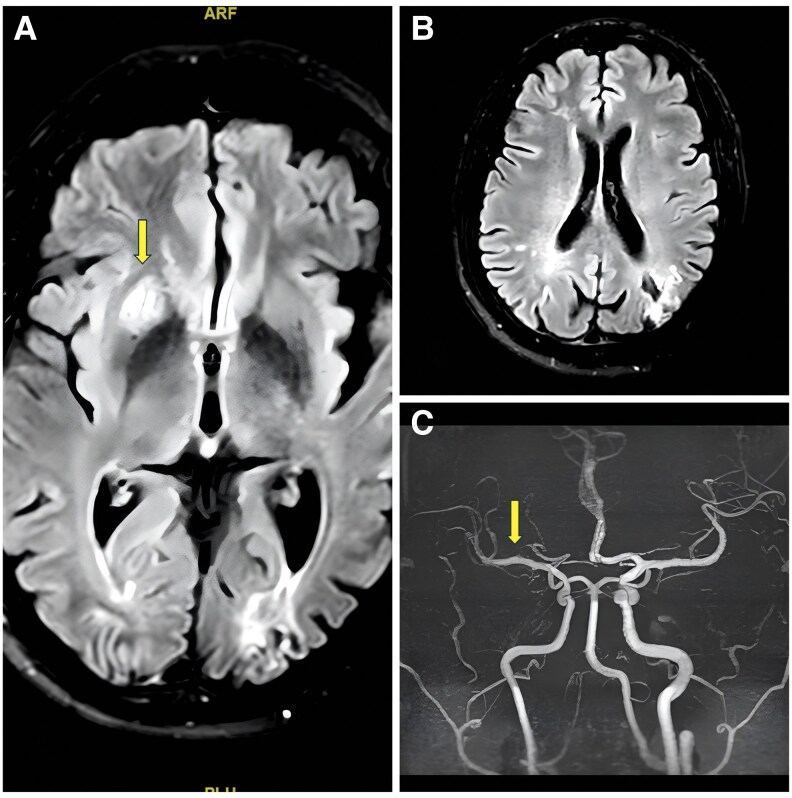
(*A*) Magnetic resonance imaging of the brain with FLAIR images of the patient showing hyperintensity in right-sided caudate and putamen suggestive of an acute infarct (shown by long arrow). (*B*) The magnetic resonance imaging of the brain of the patient also shows a bilateral old parieto-occipital infarct with gliosis. (*C*) Magnetic resonance angiography of the patient showing right-sided paucity of M3 branches of middle cerebral artery (MCA) (shown by long arrow).

A 67-year-old woman presented with slurred speech, left-sided weakness, vomiting, and pre-syncope. Her medical history includes hypertension (telmisartan 40 mg, hydrochlorothiazide 12.5 mg daily) and paroxysmal AF (bisoprolol 5 mg, flecainide 100 mg twice daily, apixaban 5 mg twice daily). She was stable on this regimen for 2 years without prior stroke, myocardial infarction (MI), or ventricular arrhythmias. On examination, she was drowsy with left-sided weakness, an irregular heart rate of 88 b.p.m., and blood pressure (BP) of 156/98 mmHg. Cardiorespiratory examination was unremarkable. Laboratory results showed normal complete blood counts and renal function, with serum values of potassium 3.96 mEq/L, calcium 9.1 mg/dL, magnesium 1.8 mg/dL, sodium 138 mEq/L, and creatinine 1.1 mg/dL. An electrocardiogram (ECG) showed an irregular narrow complex QRS wave with absent P waves, indicating AF at a controlled ventricular rate of 90 b.p.m. and a QTc interval of 495 ms (*Figure 1*). Magnetic resonance imaging (MRI) of the brain with FLAIR images revealed hyperintensity in the right-sided caudate and putamen, suggestive of acute infarct (*Figure 2*). She was admitted to stroke intensive care unit and treated for probable cardioembolic ischaemic stroke.

**Figure 3:**
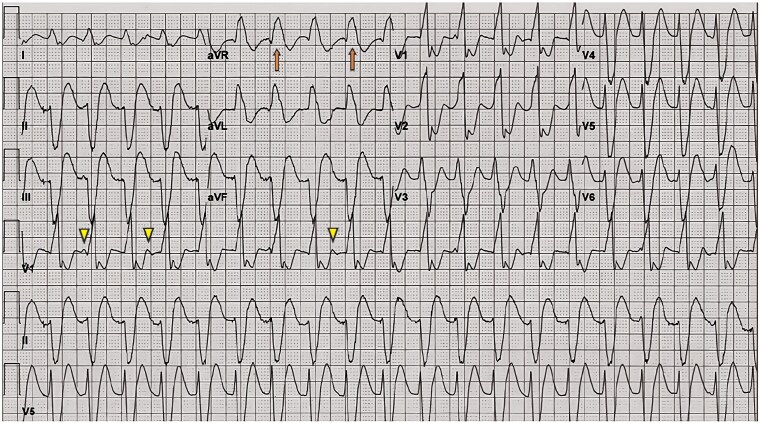
An electrocardiogram of the patient showing broad complex tachycardia at a rate of approximately 124 b.p.m., with a QRS duration of about 160 ms, atrioventricular dissociation (shown by arrowhead), extreme left axis deviation, and a positive R wave in lead augmented vector right (aVR) (shown by long arrow) and distortion of the ST segment, confirming the diagnosis of ventricular tachycardia.

**Figure 4:**
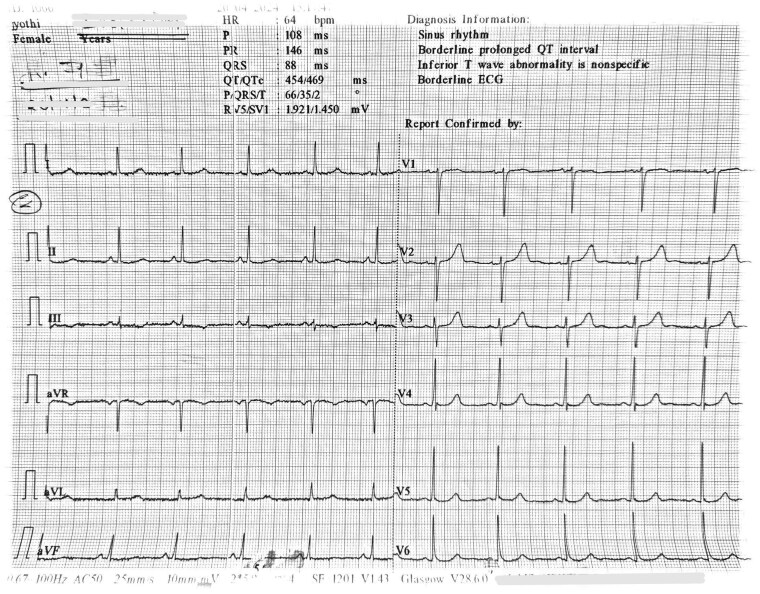
An electrocardiogram of the patient 4 h after intravenous sodium bicarbonate bolus and infusion. The tachycardia resolved within 30 min, reverting to sinus rhythm, as shown by the presence of regular P wave, and her QRS duration progressively narrowed to normal (88 ms) over 4 h.

**Figure 5:**
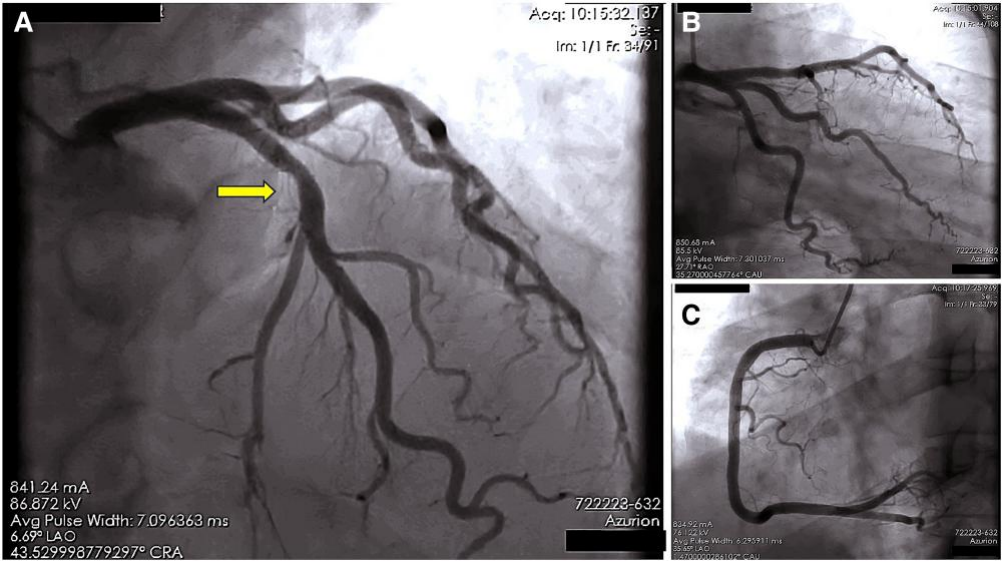
(*A*) Coronary angiogram of the patient in anteroposterior cranial projection showing Type III left anterior descending artery having minor non-obstructive plaque (shown by long arrow) in its proximal part. (*B*) Coronary angiogram of the patient in anteroposterior caudal projection showing normal left circumflex artery and obtuse marginal branch and left anterior descending artery with similar findings. (*C*) Coronary angiogram of the patient in left anterior oblique projection showing normal right coronary artery with no significant lesion.

Two days later, she experienced sudden palpitations and dyspnoea with tachycardia. Her systolic BP dropped to 90–100 mmHg, but oxygen saturation remained at 99%. The repeat ECG showed broad complex tachycardia at a rate of approximately 124 b.p.m., with a QRS duration of about 160 ms (*Figure 3*). Echocardiography revealed concentric left ventricular hypertrophy, normal left ventricular ejection fraction, and no clots or pulmonary arterial hypertension. Differential diagnosis at this stage included atrial flutter with 1:1 AV conduction due to flecainide or VT. Further ECG analysis showed wide QRS complexes with AV dissociation, extreme left axis deviation, and a positive R wave in lead augmented vector right (aVR) and distortion of the ST segment, confirming the diagnosis of VT (*Figure 3*). After two failed 200J synchronized direct current cardioversion (DCC) attempts for tachycardia, IV amiodarone (300 mg) and magnesium (2 g) were administered. A third unsuccessful DCC prompted 1.5 mg/kg IV lidocaine, which only partially reduced the rate. Suspecting flecainide-induced VT, IV sodium bicarbonate (100 mEq bolus, then 15 mEq/hour in D5W) was given with ABG monitoring to maintain pH ∼7.5. Tachycardia resolved within 30 min, restoring sinus rhythm, with QRS narrowing to 90 ms over 4 h (*Figure 4*). Her repeat blood tests revealed hyponatremia with serum sodium level of 123 mEq/L (probably due to mannitol use), which improved with IV hydration using 0.9% normal saline and increased oral salt intake. Her flecainide level was slightly elevated at 1.2 µg/mL (normal therapeutic range: 0.2–1.0 µg/mL). A repeat ECG 2 days later showed normal findings with a QRS duration of 88 ms and sinus rhythm. Coronary angiogram and cardiac MRI confirmed non-obstructive coronary artery disease and normal biventricular function without structural heart disease, indicating that the ventricular arrhythmia was likely drug-induced (*Figure 5*). Flecainide was discontinued, and she was started on amiodarone (400 mg daily) along with her other medications. She remained arrhythmia-free and stable with near-normal neurological status at last follow-up after 3 months.

## Discussion

### Mechanism of flecainide-induced ventricular tachycardia

Flecainide’s side effects arise from its potent sodium channel-blocking action, resulting in delayed conduction through the AV node and ventricles. This leads to serious complications, including AV block, ventricular tachycardia, ventricular fibrillation, and asystole on ECG. Echt and Ruskin^4^ noted that in the setting of rapid ventricular rates, flecainide’s ability to delay depolarization can predispose to re-entrant arrhythmias, as slowed conduction allows for the formation of functional blocks. Di Grande *et al*.^5^ had described a 64-year-old male with paroxysmal atrial fibrillation who developed wide complex tachycardia while on flecainide; his arrhythmia was resistant to DCC but was successfully resolved with intravenous sodium bicarbonate. Similarly, Khatiwada *et al*.^6^ reported a 67-year-old female who experienced transient cardiomyopathy and left bundle branch block due to flecainide, which improved after discontinuation of drug. Electrolyte abnormalities, such as hyponatremia, may enhance the inhibitory effect of flecainide on cardiac sodium channels. Flecainide toxicity can show up even if serum levels are normal, especially if there are issues with electrolytes, like hyponatremia. Khavandi and Walker^7^ reported a case of flecainide toxicity associated with hypokalaemia and hyponatremia. Suspected cause of these metabolic derangements was thiazide diuretics, leading to flecainide toxicity due to hyponatremia. In our case also, using mannitol along with thiazide diuretics could have produced similar effects. Emerging evidence highlights that brain–heart axis dysregulation in acute ischaemic stroke involves complex interactions between sympathetic hyperactivity and systemic inflammation, with lesion-specific effects (e.g. insular cortex damage disrupting autonomic control and triggering arrhythmias) exacerbating cardiovascular complications.^8^

### Management of flecainide-induced ventricular tachycardia

The cornerstone of treatment for flecainide-induced VT is reversal of sodium channel blockade, which can be achieved with intravenous sodium bicarbonate. Sodium bicarbonate increases extracellular concentration of sodium ions, displacing flecainide from its binding sites on sodium channels and thereby restoring normal conduction. Additionally, sodium bicarbonate raises serum pH, which decreases the availability of the drug in its active form. Brugada *et al*.^2^ had found that sodium bicarbonate is more effective than other antiarrhythmics, such as lidocaine, in reversing effects of flecainide-induced sodium channel blockade. Kasia *et al*.^9^ also described the treatment of flecainide-induced VT in a patient with a pacemaker using sodium bicarbonate. Other therapeutic strategies for managing flecainide-induced arrhythmias include the use of lidocaine, a Class IB antiarrhythmic. While lidocaine acts by blocking inactivated sodium channels with faster kinetics than flecainide, its efficacy is limited, and it can further slow conduction in cases of flecainide toxicity, making its use typically avoided. Beta-blockers may serve as adjunctive therapy by reducing heart rate and allowing more time for sodium channels to recover from blockade caused by flecainide. In this case, the patient’s bisoprolol use likely helped maintain stable haemodynamics during the VT episode. Khatiwada *et al*.^6^ showed that intravenous fat emulsion therapy can effectively treat flecainide toxicity. There are no established guidelines for flecainide level monitoring since toxicity can occur even within the normal range values. We recommend, in case there is any electrolyte imbalance or QTc changes, that the flecainide level should be checked. Values towards the upper limit of the reference range or even a slight increase from the same should prompt heightened active vigilance for the development of any arrhythmia and corrections for these precipitating factors. Also, key strategies should include readiness to administer sodium bicarbonate for wide complex tachycardia in these scenarios.

## Lead author biography


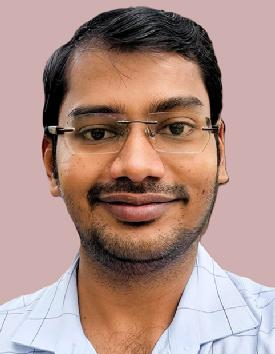


Dr Abhishek Kumar, with a Doctor of Medicine (DM) degree in Cardiology, is working as an Assistant Professor at the Department of Cardiology, Government Superspeciality Hospital CIMS, Koni, Bilaspur, India, which is a government tertiary healthcare and research institute. His research focuses on critical areas such as acute heart failure, high-sensitivity cardiac troponins, and strain echocardiography, contributing valuable insights to the field with multiple published papers. His clinical expertise includes proficiency in primary angioplasty, pacemaker implantation, and structural cardiac interventions, making him a well-rounded practitioner.

**Consent:** The authors have received written consent from the patient for this case report, including images, following the COPE guidelines.

**Funding:**. None declared.

## Data Availability

The data underlying this article are available in the article and in its online supplementary material.
